# Words of Courage

**Published:** 1881-11

**Authors:** 


### WORDS OF COURAGE.

A GREAT deal of talent is lost to the world for the want of a little courage. Every day sends to the grave a number of obscure men who have only remained in obscurity because their timidity has prevented their making the first effort, and who, if they could have been induced to begin, would in all probability have gone great lengths in the career of fame. The fact is, in order to do anything in this world that is worth doing, we must not stand shivering on the brink, and think of the cold and danger, but jump in and scramble as we can. It will not do to be perpetually calculating risks and adjusting nice chances. It did very well before the flood, when a man could consult his friends upon a publication for one hundred and fifty years, and then live to see its successes six or seven centuries afterward; but at present a man waits and doubts, and hesitates, and consults his brother, and his uncle, and his particular friends, till one day he finds that he is sixty years of age; that he has lost so much time in consulting first cousins and particular friends that he has no time left to follow their advice. There is such little time for over-squeamishness at present, the opportunity so easily slips away, the very period of his life at which man chooses to venture, if ever, is so confined, that it is no bad rule to preach up the necessity, such instances, of a little violence done to feelings, and of efforts made in defiance of strict and sober calculation.

